# Loss of the Nuclear Receptor Corepressor SLIRP Compromises Male Fertility

**DOI:** 10.1371/journal.pone.0070700

**Published:** 2013-08-15

**Authors:** Shane M. Colley, Larissa Wintle, Richelle Searles, Victoria Russell, Renee C. Firman, Stephanie Smith, Kathleen DeBoer, D. Jo Merriner, Ben Genevieve, Jacqueline M. Bentel, Bronwyn G. A. Stuckey, Michael R. Phillips, Leigh W. Simmons, David M. de Kretser, Moira K. O'Bryan, Peter J. Leedman

**Affiliations:** 1 Laboratory for Cancer Medicine, The University of Western Australia Centre for Medical Research, Western Australian Institute for Medical Research, Perth, Australia; 2 Research Centre, Royal Perth Hospital, Perth, Australia; 3 Centre for Evolutionary Biology, School of Animal Biology, The University of Western Australia, Crawley, Australia; 4 Male Infertility and Germ Cell Biology Laboratory, Department of Anatomy and Developmental Biology, Monash University, Clayton, Australia; 5 Keogh Institute for Medical Research, Sir Charles Gairdner Hospital, Nedlands, Australia; 6 Anatomical Pathology, PathWest Laboratory Medicine, Royal Perth Hospital, Perth, Australia; 7 School of Pathology and Laboratory Medicine, University of Western Australia, Crawley, Australia; 8 School of Medicine and Pharmacology, University of Western Australia, Crawley, Australia; Baylor College of Medicine, United States of America

## Abstract

Nuclear receptors (NRs) and their coregulators play fundamental roles in initiating and directing gene expression influencing mammalian reproduction, development and metabolism. SRA stem Loop Interacting RNA-binding Protein (SLIRP) is a Steroid receptor RNA Activator (SRA) RNA-binding protein that is a potent repressor of NR activity. SLIRP is present in complexes associated with NR target genes in the nucleus; however, it is also abundant in mitochondria where it affects mitochondrial mRNA transcription and energy turnover. In further characterisation studies, we observed SLIRP protein in the testis where its localization pattern changes from mitochondrial in diploid cells to peri-acrosomal and the tail in mature sperm. To investigate the *in vivo* effects of SLIRP, we generated a SLIRP knockout (KO) mouse. This animal is viable, but sub-fertile. Specifically, when homozygous KO males are crossed with wild type (WT) females the resultant average litter size is reduced by approximately one third compared with those produced by WT males and females. Further, SLIRP KO mice produced significantly fewer progressively motile sperm than WT animals. Electron microscopy identified disruption of the mid-piece/annulus junction in homozygous KO sperm and altered mitochondrial morphology. In sum, our data implicates SLIRP in regulating male fertility, wherein its loss results in asthenozoospermia associated with compromised sperm structure and mitochondrial morphology.

## Introduction

Infertility affects approximately 1 in 8 couples globally, with the cause being attributable to a male factor in approximately half of all cases [1]. Despite the considerable increase in our understanding of fertility, the diagnosis for many couples unable to conceive is frequently idiopathic. Determining the basis of infertility and providing effective and early screening for identified causes may lead to more effective clinical intervention.

Multiple factors determine fertility and in particular spermatogenesis [2]–[4]. It is estimated that 4% of the mouse genome encodes molecules expressed in post-meiotic male germ cells alone [5] with over 400 recombinant animal models showing alterations in fertility as a result of gene manipulation [2], [6]. Mutations in genes critical to spermatogenesis and function have been found to cause defects to functional components of sperm including mitochondria [7], [8], the acrosome [9], annulus [10] and flagellum [11].

Pivotal to the production of functional spermatozoa is an intact endocrine signalling system. Pituitary and hypothalamus-derived hormones along with nuclear hormones produced within the testis, particularly testosterone, are obligate regulators of this process [12]. Perturbation of hormone synthesis, secretion, interaction with their receptors or the activities of their cognate coregulatory machinery may result in partial or complete infertility [13].

NR coregulators, the bridging apparatus between NRs and the transcriptional machinery, co-ordinately regulate NR function in a ligand-dependent manner, by coactivating (e.g. SRC-1) or corepressing (e.g. NCoR) target gene expression [14]. Although there is increasing evidence that aberrant coregulator activity may contribute to infertility, we still know relatively little about the function of these coregulators in sperm, and none to date have been identified as RNA-binding proteins. What is known however is that SRC-1, which is localized to Sertoli cells and spermatocytes [15], is crucial for fertility as SRC-1 KO mice are sub-fertile, with impaired spermatogenesis [16]. In addition, inactivation of SRC-2, a coactivator of the androgen receptor, results in sub-fertility characterized by defects in both spermiogenesis and age-dependent testicular degeneration [17]. Additionally, over-expression of SRA, the only NR coregulator that acts as an RNA [18], [19] results in germ cell aplasia and compromised male fertility [20].

SLIRP is a SRA-binding NR coregulator that acts as a potent corepressor of multiple NRs including the androgen and estrogen receptors [21], [22]. SLIRP is recruited to NR-regulated promoters in hormone responsive tissues, consistent with it playing an important role modulating steroid hormone action. In normal human tissue, SLIRP is most highly expressed in skeletal muscle, heart, liver and the testes [21]. Further, although SLIRP is present in the nucleus and associated with NR promoters, it is also present in the mitochondria. SLIRP has been shown to regulate mitochondrial RNA expression [23] and influence oxidative phosphorylation [24], [25]. Based on SLIRP's expression in human testes and its regulation of transcription, we explored its role in male reproductive function by more closely investigating its expression in WT mice and by creating a SLIRP KO mouse. We found that SLIRP is present within male germ cells and that its localization alters during spermatogenesis, that male SLIRP KO mice are sub-fertile and have poor sperm motility associated with abnormalities of the distal mid-piece and positioning of the annulus of their gametes. These data provide new insights into the role of NR coregulators in fertility and suggest a novel role for SLIRP in spermatogenesis.

## Results

### SLIRP in Developing and Mature Spermatozoa

**Figure 1:**
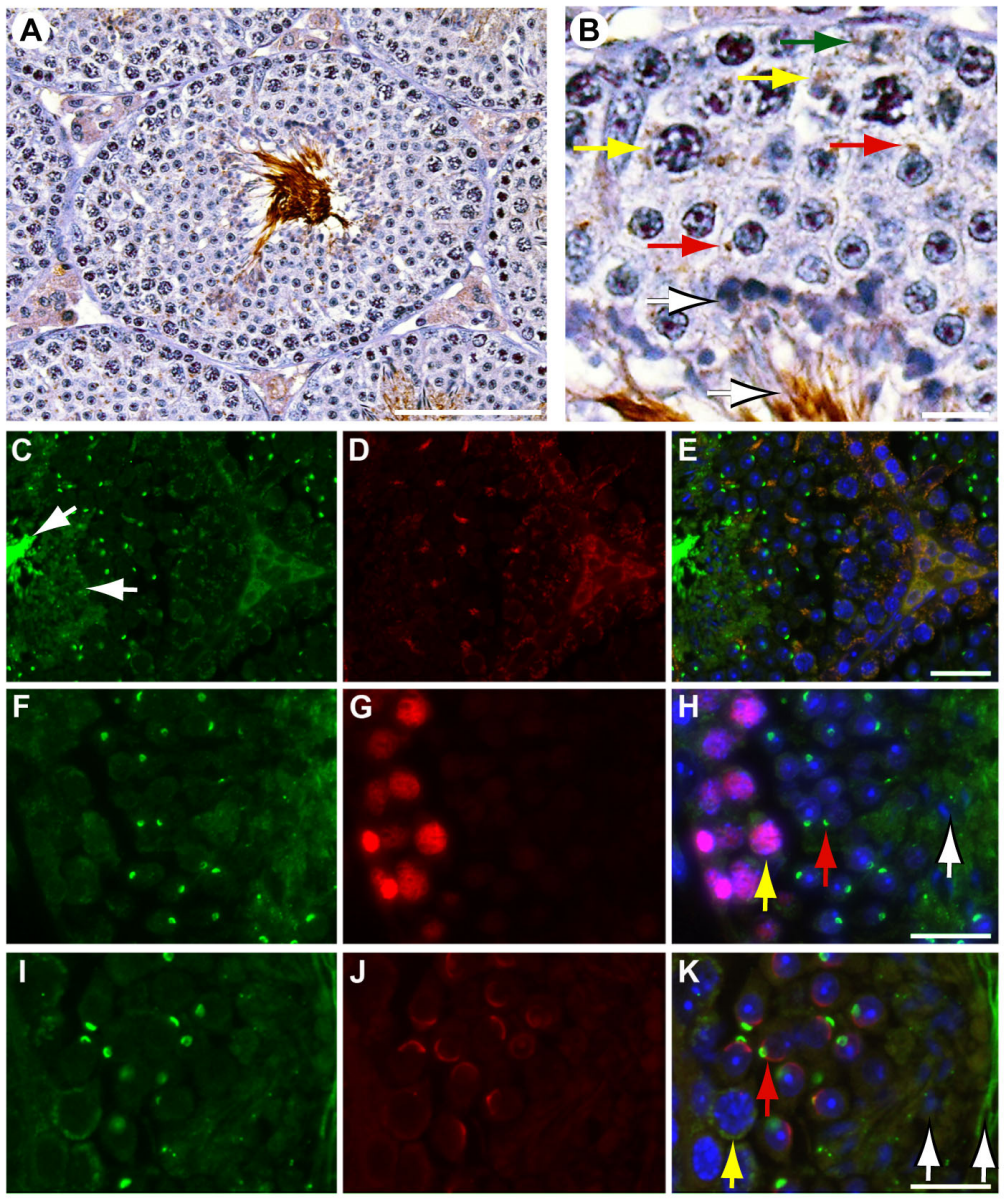
SLIRP is expressed in the mouse testis. (A & B) Detection of SLIRP by immunohistochemistry in Leydig cells, spermatogonia (green arrow), early spermatocytes (yellow arrows), round spermatids (red arrows), elongate spermatids and sperm lining the lumen (white arrows). (C, F, I) Immunofluorescent staining for SLIRP (green), (D) Hsp60 (red), PCNA (G, red) and SP56 (J, red) overlayed in E, H & K respectively. Yellow indicates colocalization in overlayed panels. Nuclear DAPI staining in blue E, H & K. White bars: A, 80 µm, B, H & K, 10 µm, E, 20 µm.

To define SLIRP localization in adult male mice, we performed immunohistochemistry on testis sections from WT animals (Fig. 1A & B). SLIRP protein is localized within the cytoplasm of Leydig cells associated with seminiferous tubules, diploid spermatogonia (green arrow) and in pre-leptotene through to pachytene spermatocytes (yellow arrows also Fig. 1H & K). Within the latter it has a peri-nuclear, crescentic distribution. In spermatids, SLIRP staining is more condensed and lies adjacent to the developing acrosome (red arrows, also Fig. 1H & K). Within elongate spermatids, SLIRP is present in the tail and is diffusely cytoplasmic (white arrows; also Fig. 1C, H, K & S1).

Immunofluorescent staining of testis sections for SLIRP (Fig. 1C) and the mitochondrial marker Hsp60 (Fig. 1D) showed co-localization in the inter-tubular tissue and spermatogonia similar to its localization in cell lines as previously reported (Fig. 1E, 21). Within primary spermatocytes, SLIRP is localized with the cytoplasm overlapping with Hsp60. However, in more mature germ cells, this co-localization is lost with no evidence of overlay in elongate spermatids. Further highlighting this change in distribution of SLIRP with maturation, SLIRP (Fig. 1F) is weakly cytoplasmic in proliferating PCNA-positive spermatogonia-leptotene spermatocytes (Fig. 1G, yellow arrow) but its staining is more condensed in more differentiated PCNA-negative cells (Fig. 1H, red arrow).

**Figure 2:**
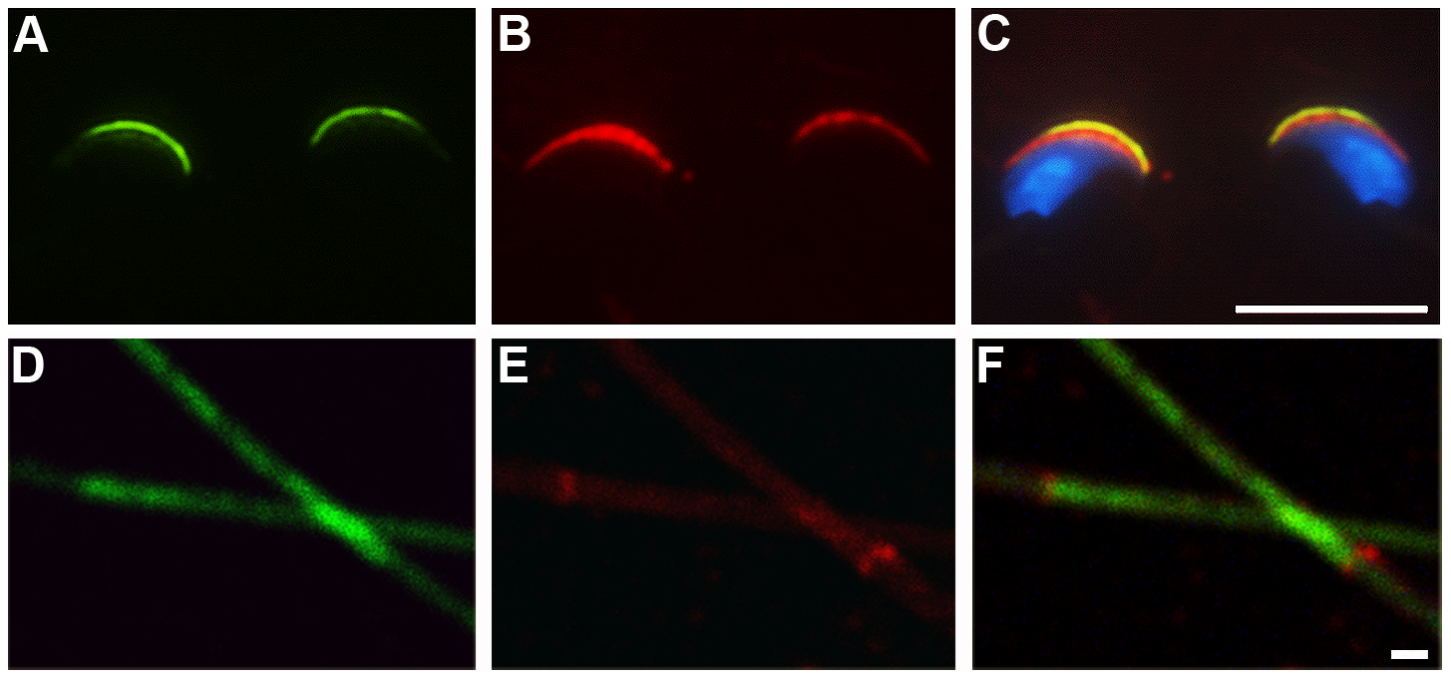
SLIRP localization in epididymal sperm. (A) Immunofluorescent staining for SLIRP (green) and Sp56 (B, red) in sperm heads (C, overlay). (D) SLIRP (green) and Septin 4 (E, red) staining of sperm tails (F, overlay). White bars: C, 10 µm; F, 1 µm.

In early round spermatids, immunofluorescent staining for SLIRP (Fig. 1I) is localized adjacent to the acrosome marker SP56 (Fig. 1J) indicating a peri-acrosomal distribution (Fig. 1K). Consistent with these observations, within epididymal sperm SLIRP is present in the rostral acrosomal area of the mature sperm (Fig. 2A) and partially overlaps with SP56 (Fig. 2B, overlay 2C). Staining for Septin 4, an annulus component (Fig. 2E), shows that while SLIRP (Fig. 2D) is present in the sperm tail, it does not appear to be associated with the annulus protein (overlay Fig. 2F). Taken together these data indicate that SLIRP localization alters from primarily mitochondrial in spermatogonia to peri-nuclear crescentic in spermatocytes and finally to peri-acrosomal and tail localized in mature sperm.

### Generation of the SLIRP Knockout mouse

**Figure 3:**
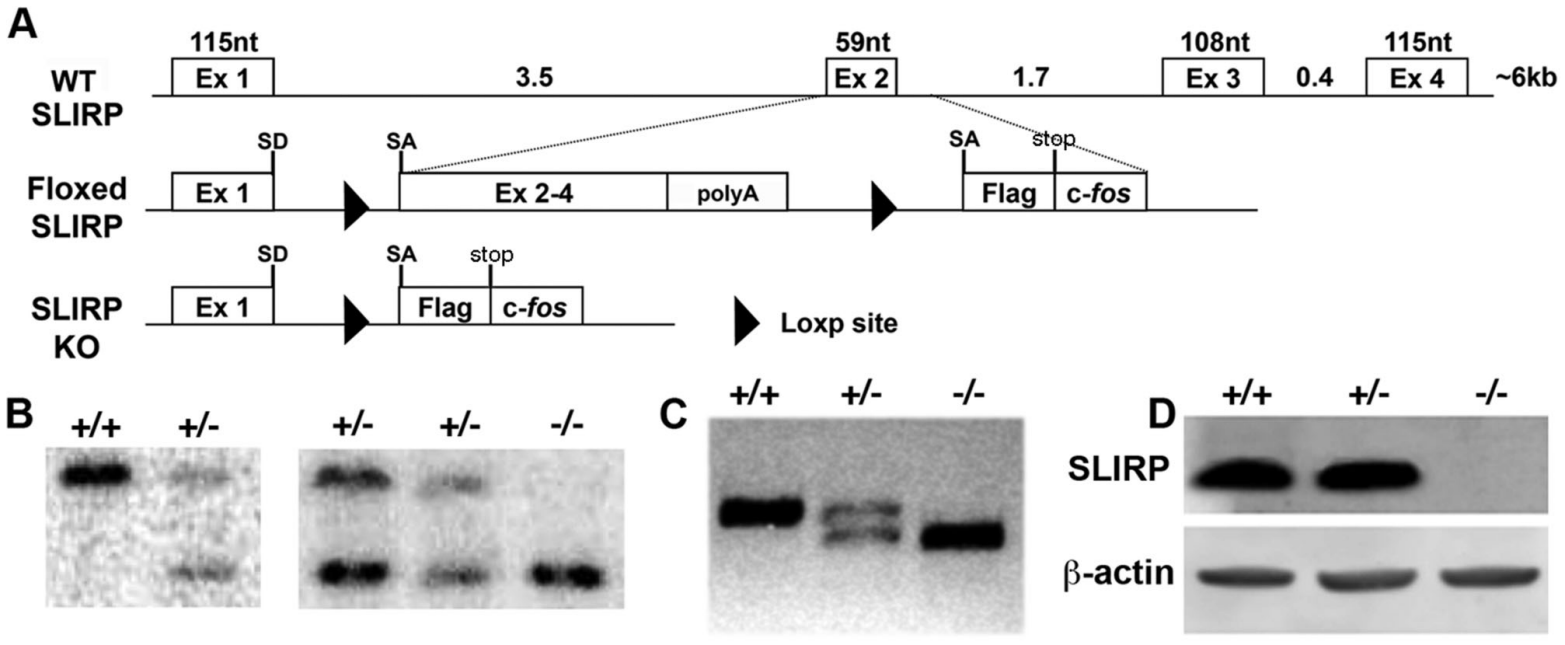
Generation of the SLIRP KO mouse. (A) Wild type (WT), floxed SLIRP and SLIRP knockout (KO) mouse SLIRP locus configurations. Floxed mice contain a cDNA for SLIRP exons 2 to 4 (Ex 2–4) with polyadenylation signal (poly A) preceded by loxp and splice acceptor (SA) sites and followed by loxp, SA, Flag epitope, stop codon and c-*fos* sequences inserted within the first SLIRP intron (intron length in kb, nt, nucleotides). (B) Southern analysis to detect a 31.3 kb EcoRV fragment in WT (+/+) and heterozygous (+/−) mice and a 13.3 kb recombinant band in heterozygous and SLIRP homozygous KO (−/−) animals. (C) WT (334 nt) and recombinant (247 nt) RT-PCR products generated from WT and recombinant SLIRP mouse liver cDNAs. (D) Western analysis of WT and recombinant SLIRP mouse testis lysates for SLIRP and β–actin.

To assess the *in vivo* effects of deleting the SLIRP gene, we generated a conditional SLIRP KO mouse. The murine SLIRP gene (also referred to as RIKEN cDNA 1810035L17, NCBI Reference Sequence: NM_026958.3) is composed of 4 exons (Fig. 3A) and is located on chromosome 12 (12E). At its 5′ end, the SLIRP gene is adjacent to ALKBH1, which is in the opposite transcriptional orientation, with the transcript initiation sites predicted to be separated by 59 bases. As a result, a deletion strategy to remove the SLIRP gene while minimising disruption to non-targeted sequences was employed (see methods). KO SLIRP mice were generated by crossing the floxed SLIRP mouse with a pan-tissue cre expressing animal thus deleting the introduced exon 2 to 4 cDNA resulting in the remaining exon 1 containing transcripts being spliced to an artificial exon containing an inframe Flag epitope, stop codon and c-*fos* mRNA instability sequence. Southern analysis confirmed the generation of the SLIRP KO mouse, with WT and heterozygous KO mice demonstrating a 31.3 kb WT SLIRP EcoRV digestion fragment while a 13.3 kb recombinant sequence was present in heterozygous and KO mouse material (Fig. 3B). RT-PCR performed using a combination of primers homologous to exon 1, exon 4 and Flag/*fos* bearing transcripts showed a single 334 nt product for WT mice, together with a 247 nt recombinant RNA specific amplicon in the heterozygotes, the latter being the sole product in the KO sample (Fig. 3C). These data were further confirmed by western analysis demonstrating the presence of SLIRP protein in WT and heterozygous animals but not homozygous KO mice (Fig. 3D). Sequencing of the RT-PCR products confirmed recombination had occurred as designed while Flag-tagged residual protein levels were below that detectable by western analysis (data not shown). Further, qRT-PCR for ALKBH1 and SKIP mRNA confirmed there was no significant difference in the transcription of these genes which flank SLIRP between the genotypes (Fig. S2).

### Homozygote SLIRP KO pups are under represented in heterozygous matings

Genotyping of pups resulting from heterozygous SLIRP KO mouse breeding revealed that homozygous offspring were under represented compared with heterozygous and WT pup proportions as predicted by Mendelian genetics. In assessing 775 pups reaching weaning age resulting from heterozygote matings, 190 WT (25%), 419 heterozygous (54%) and 166 homozygous KO (21%) mice were identified. One Sample Test of Proportion analysis of these data confirms there was a significant reduction in homozygous KO pups produced compared with the predicted 25% (p = 0.02). These outcomes indicate that while SLIRP is not absolutely required for life, its absence is associated with a reduction in SLIRP KO pup production compared with WT mice.

### SLIRP KO Males Produce Smaller Litters and their Sperm are Less Progressively Motile

**Figure 4:**
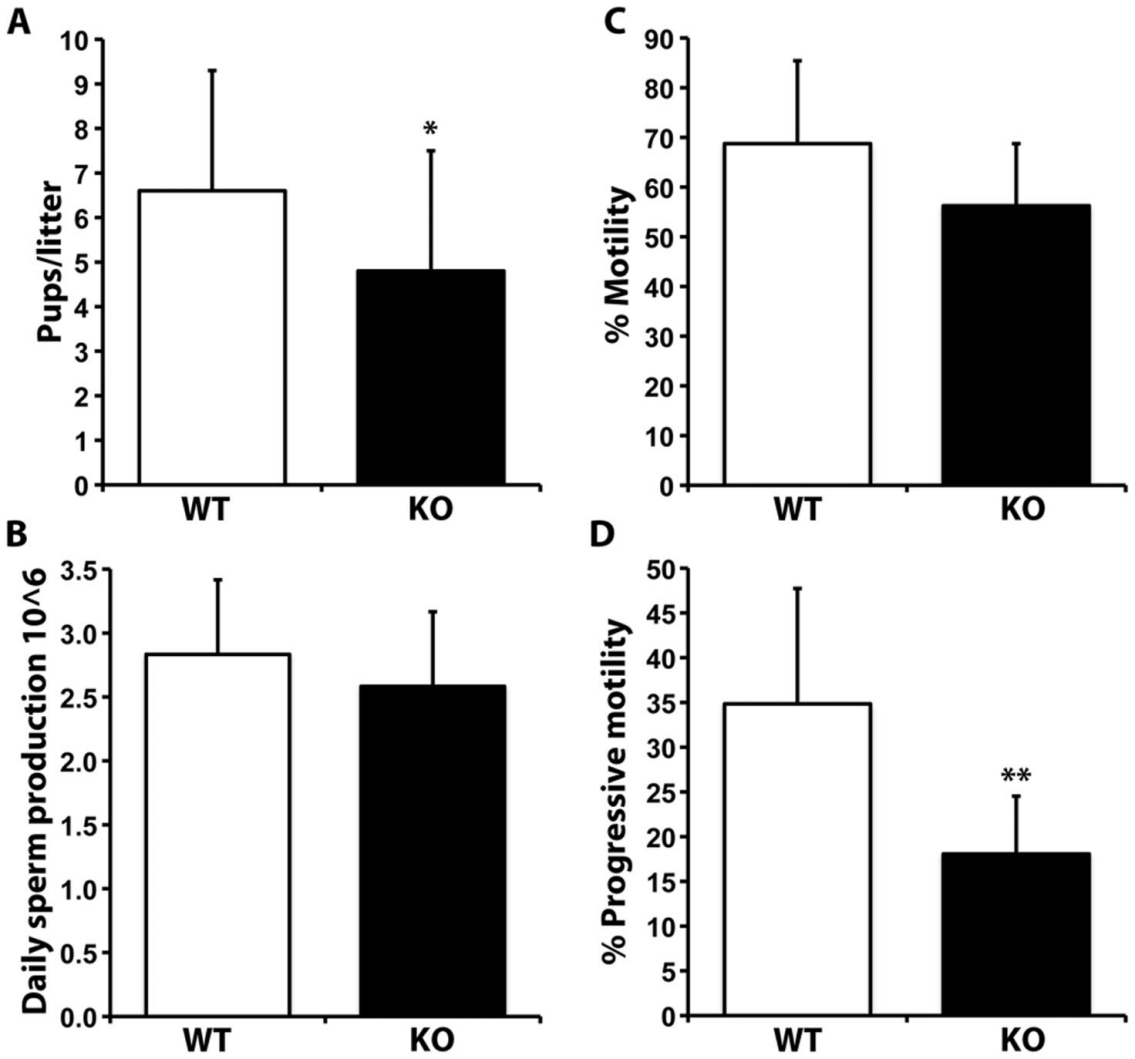
SLIRP KO males produce smaller litters and have fewer progressively motile sperm. (A) Litter sizes resulting from breeding wild type (WT) females with WT or SLIRP knockout (KO) males (*, significant difference by Maximum Likelihood analysis of repeated measures, p = 0.025). (B) Comparison of daily sperm production (B), motility (C) and progressive motility (D) between WT and SLIRP KO animals. **, ANOVA, p<0.001.

Subsequent breeding with homozygous SLIRP KO males revealed they produce fewer pups than their WT counterparts. WT males bred with WT females produce an average of 6.9 (sd 2.7) pups per litter compared with KO males with WT females that produce only 4.5 (sd 2.4; Fig. 4A; 5WT males bred with 9 WT females for 20 litters, 7KO males bred with 7 WT females for 12 litters). Using Maximum Likelihood analysis of repeated measures it was determined that the approximately one third reduction in litter size resulting from SLIRP KO males is significant (p = 0.025) and, consistent with its localization in the testis and sperm (Figs. 1 & 2), points to SLIRP playing an important functional role in this tissue and fertility.

To investigate why male SLIRP homozygous KO mice produce smaller litters their sperm production and function was compared with that from WT counterparts. Daily sperm production and testis weights were not different between the WT and KO male mice (Fig. 4B; 15 WT and 17 KO males compared). This is consistent with no difference being observed in testis histology by light microscopy or serum testosterone, follicle stimulating or luteinizing hormone levels between genotypes (Fig. S3 & S4). Subsequently computer assisted sperm analysis (CASA) found total motility (any form of movement) was not significantly different between genotypes (Fig. 4C), however, sperm from SLIRP KO mice have a significantly decreased capacity for progressive motility (18.8%) compared to their WT littermates (34.4%; Fig. 4D, Fig. S5 and Movie S1; Fig. S6 and Movie S2). As progressive motility is an absolute requirement for fertility the reduced levels in SLIRP KO sperm is likely to contribute to the observed decrease in litter size. Given their normal levels of sperm production but reduced progressive motility, these mice may be considered asthenozoospermic.

### SLIRP KO Sperm have Aberrant Annulus and Mitochondrial Structures

**Figure 5:**
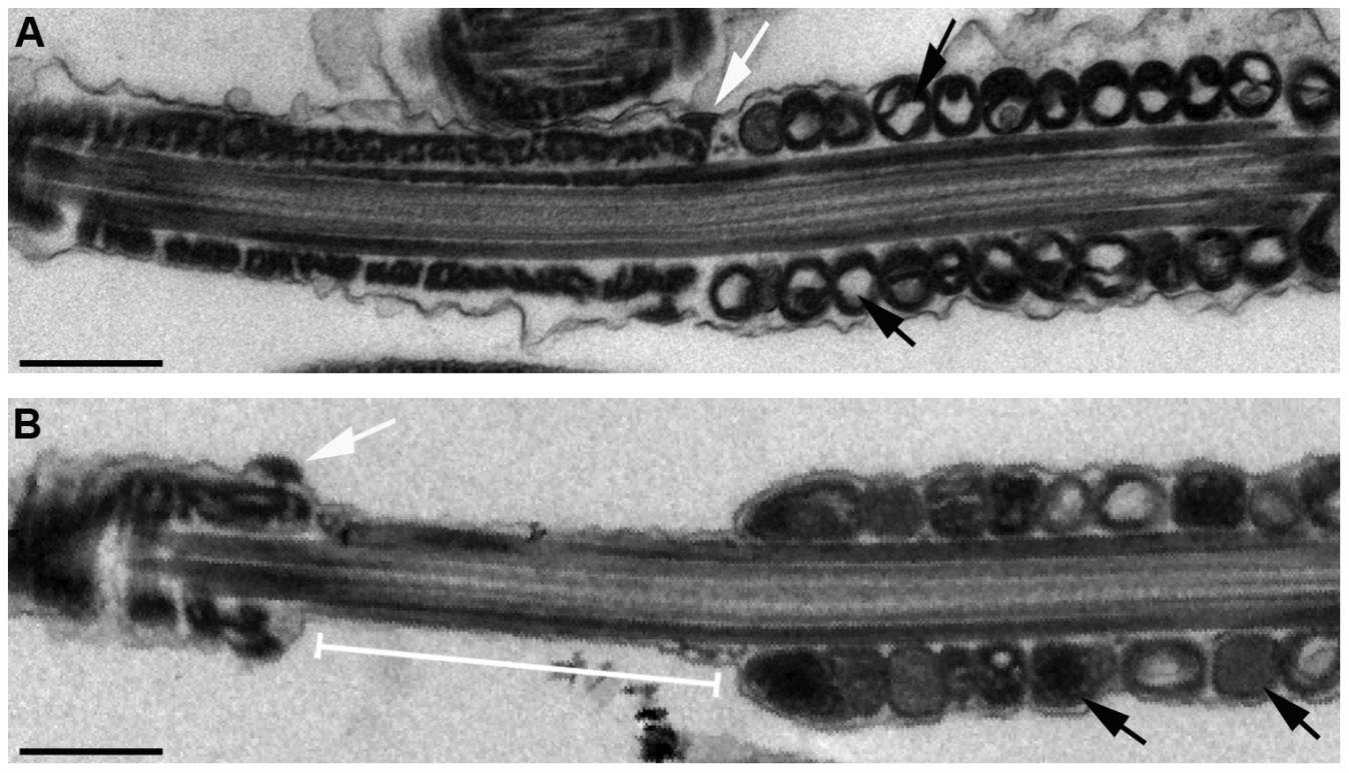
Annulus and mitochondrial disruption in SLIRP KO sperm. Electron micrographs of the distal mid-piece and annulus region of (A) WT and (B) SLIRP KO sperm. White arrows, annulus; white bar, abnormal mid-piece/annulus junction in KO sperm; black arrows, electron light and dense areas in WT and KO mitochondria respectively. Black bars, 0.5 µm.

Electron microscopy revealed significant abnormalities between WT and SLIRP KO sperm (Fig. 5 and S7). The mitochondrial cisternae in KO sperm appear less defined and the mitochondrial matrix more electron dense than in WT samples. Significantly, compared to the close apposition of the termination of the mitochondrial sheath of the mid-piece and the annulus in WT sperm (Fig. 5A), KO sperm almost uniformly contained a gap between these structures (Fig. 5B). Together these data indicate that SLIRP is required for the normal formation of the sperm mid-piece and that its loss leads to mitochondrial and annular abnormalities that may together or separately result in the observed poor progressive motility of SLIRP KO sperm and ultimately their reduced fertility.

## Discussion

Data presented herein demonstrate that SLIRP is produced in murine testes and co-localizes to mitochondria in spermatogonia and early spermatocytes before shifting to peri-acrosomal in spermatids and to the acrosomal region and tail of mature sperm. Further, SLIRP deletion is associated with male sub-fertility characterized by the production of sperm with reduced progressive motility, abnormalities in mitochondrial structure and the disjunction of the mitochondrial sheath and annulus. This is associated with homozygous KO mice being under represented in litters resulting from heterozygous matings. While SLIRP has previously been demonstrated to function as an SRA-binding NR corepressor and regulator of mitochondrial activity, we show that it is required for the structural integrity of spermatozoon, sperm motility and ultimately fertility.

Whilst there are many causes for impaired sperm motility in murine infertility models [2], [6], this is the first evidence, to our knowledge, of a NR coregulator abnormality producing a structural defect. Electron microscopy identified aberrant mitochondrial structures in SLIRP KO sperm consistent with its localization to mitochondria in spermatogonia and primary spermatocytes early in spermatogenesis, suggesting SLIRP plays an important role in the biology of this organelle. This adds to reports that SLIRP impacts on oxidative phosphorylation and mitochondrial gene expression [24], [25]. Optimal mitochondrial function is essential for fertility as sperm require energy for survival and motility and during germ cell maturation, mitochondria undergo morphological and subcellular organisational change. Dysfunction of the mitochondrial electron transport chain (ETC) in post-meiotic spermatogenesis has been associated with abnormal mitochondrial membrane potential and oxidated stress resulting in various pathologies including DNA damage (mitochondrial and nuclear), incomplete repair of oxidative phosphorylation enzymes, protein damage, lipid peroxidation and loss of calcium stores giving rise to oligoasthenozoospermia, reduced sperm function and decreased pregnancy rates [26]–[28]. Given that SLIRP can regulate a select number of mitochondrial genes [24] it is interesting to consider that its absence is associated with structural and morphological changes in sperm. It is notable that mitochondrial oxidative phosphorylation is not an absolute requirement for hyperactivated sperm motility in mice while glycolysis is [29]. Thus, although loss of SLIRP is associated with reduced progressive motility, KO sperm still retain the ability to fertilise ova but possibly with reduced efficiency due to compromised energy production/utilization.

In addition to the mitochondrial defects revealed by EM, a pronounced disruption of the distal segment of the mid-piece and its junction with the annulus was identified in SLIRP KO sperm. As well as acting as a barrier separating the mid and principal pieces of sperm, the annulus facilitates mitochondrial migration, organisation and alignment [30]. A number of annular proteins have been identified including Septins 4 and 7 along with tat1, the absence of which have been associated with impaired sperm motility in both humans and mice [30]–[33]. Paralleling the changes observed with localisation of SLIRP in mouse testis, DNAJB13 associates with Septin 4 during spematogenesis but is absent from the mature gamete indicating a role in annulus assembly [34]. Within mouse sperm, SLIRP is localized to the peri-acrosomal region, along the mid- and principal pieces, but we were unable to identify it in the annulus *per se*. The cytoplasmic localization of SLIRP in elongating spermatids at the final stages of spermiogenesis is likely to represent its presence in the residual body shed prior to the release of the mature sperm.

Multiple RNA-binding proteins have been identified within the testis residual body that function to regulate transcription and translation, the targeted deletion of which result in a range of infertility phenotypes [35]. Given SLIRP's ability to bind RNA and affect both nuclear [21] and mitochondrial gene transcription [25] it is tempting to speculate that its interaction with discrete RNAs within the testis may affect the correct assembly of the mid-piece/annulus region. Notably, testis over expression of SRA, the first target identified for SLIRP binding [21], is associated with smaller litter size and germ cell aplasia in the testis [20]. Taken together, these data suggest that SLIRP plays an important role in maintaining the integrity of mitochondria, sperm morphology and motility.

Our *in vivo* finding that loss of SLIRP in mice results in sub-fertility and compromised sperm morphology is highly suggestive of a role in humans. We postulate that men with absent or decreased SLIRP expression would have reduced fertility, reduced progressive sperm motility and potentially abnormal mitochondrial function and structure. A gene array study comparing the sperm RNA expression profiles between men of normal fertility and teratozooospermic individuals found that reduced SLIRP levels were a frequent observation in the latter group [36].

The studies presented provide novel insights into the *in vivo* role of SLIRP in the testis and sperm. Deletion of this RNA-binding NR corepressor from the murine genome is associated with disjunction of the distal portion of the sperm mid-piece from the annulus, altered mitochondrial morphology within sperm and their reduced progressive motility. The resultant asthenozoospermia within these mice points to a similar loss leading to reduced fertility in humans.

## Materials and Methods

### Animals

Animal studies described herein were performed in accordance with the National Health and Medical Research Council of Australia's Code of Practice for the Care and Use of Animals for Scientific Purposes and with approval from the University of Western Australia and Monash University's Animal Ethics Committees. Mice were housed in specific pathogen-free facilities at 22.5°C with 12 hr light/1 hr red/10 hr dark/1 hr red cycle conditions and fed Rat and Mouse Cubes (Specialty Feeds, Australia). For litter size comparison, only crossings between females up to 27 weeks and males to 33 weeks were assessed.

### Histological Methods and Cytology

Whole testes were removed from adult males (8–26 weeks) and fixed in Bouin's fixative for 24 hr. Tissues were embedded in paraffin and 4 µm sections mounted onto Superfrost plus slides (Menzel Gläser), dewaxed, and rehydrated. Antigen retrieval was carried out by microwave irradiation at 100°C in EDTA (13 mM, pH 8.0) buffer. Tissue sections were permeabilized in 0.2% Triton X-100/PBS for 5 min, followed by 0.05% Triton X-100/PBS for 10 min. Tissue sections were treated with 3% (vol/vol) H_2_O_2_ before immunostaining using the Dako EnVision system and counterstaining with haematoxylin. Rabbit anti-mouse SLIRP polyclonal IgG was prepared against a C-terminal 20 amino acid mouse SLIRP peptide conjugated to KLH and purified from serum via protein G-Sepharose chromatography. For tissue immunofluorescent studies, PCNA (Dako, M0879), Hsp60 (Santa Cruz, SC13115), Sp56 (QED Bioscience Inc, 55101) and Septin 4 (SC20415) primary antibodies were used. For cytological staining, caudal epididymal sperm were isolated in Human Tubal Fluid and spread on superfrost plus slides. Samples were fixed in 2% paraformaldehyde/PBS and blocked in 5% fetal bovine serum for 1 hr. AF488 and AF546 (Invitrogen) fluorescent secondary antibodies were used as listed with nuclei stained with DAPI.

### Sperm Production, Motility and Electron Micrograph Assessments

Daily sperm output was determined using the Triton X100 solubilization method as described previously [37]. Sperm motility was assessed using CASA [38] and ultra-structure using electron microscopy [39].

### Generation and Validation of the SLIRP KO Mouse

The floxed SLIRP mouse was generated by inserting into the mouse genome a cDNA coding for exons 2 to 4 of SLIRP bounded at its 5′ end by loxp and splice acceptor sites and a Bovine Growth Hormone polyadenylation signal at its 3′end. Down stream of this was a neomycin selection cassette flanked by FRT sites followed by an artificial exon with a splice acceptor, Flag epitope coding sequence, stop codon and c-*fos* derived mRNA destabilisation sequence, the latter to facilitate the rapid turnover of recombinant transcripts following gene rearrangement [40]. This construct, prepared within the pFLSniper vector (Ozgene, Australia), was inserted between −37 nt and +19 nt of SLIRP exon 2 following electroporation into C57BL/6 derived stem cells [41]. Resultant neomycin resistant clones were injected into Balb/c derived blastocysts and chimeric pups arising following implantation were crossed with PGK promoter driven flp and cre expressing C57BL/6 lines to generate SLIRP^flox/flox; neo−/−^ and SLIRP^−/−^ mice respectively. Southern analysis was performed on EcoRV digested genomic DNA isolated from mice using a 3′ SLIRP locus probe. WT and recombinant SLIRP transcripts were detected by RT-PCR. In brief, total RNA was isolated from mouse tissues utilizing Trizol and cDNA prepared using Superscript II (Invitrogen) as per manufacturer's instructions. SLIRP transcripts were detected by PCR using a combination of 5′MuSLIRPex1 ATGGCAGCCTCCGCCAT plus either 3′MuSLIRPex4 CTCAGAAATCTTTCTTCATCA or 3′KOfos AGGTCGACGGTATCGATAA primers to detect WT and recombinant transcripts respectively. Western analysis was performed using rabbit-anti-mouse SLIRP, Flag (Sigma, F1804) and β-actin antibodies (Abcam, ab6276).

## Supporting Information

Figure S1 **Immunofluorescent detection of SLIRP in mouse testis.** SLIRP localisation alters from cytoplasmic in spermatogonia (arrows with round tail) to cresentic, peri-acrosomal in spermatocytes (arrows) and diffusely cytoplasmic in spermatids (asterisk).
(TIFF)

Figure S2 **ALKBH1 and SKIP transcript levels do not differ beteween SLIRP genotypes.** Liver cDNA prepared from wild type (WT), heterozygous (HET) and SLIRP knockout (KO) mice were assessed for the expression of ALKBH1 and SKIP mRNAs relative to GAPDH. No significant difference in mean expression was found between the genotypes (n = 4/genotype).
(TIFF)

Figure S3 **Wild Type and SLIRP KO testis appear histologically similar.** Haematoxylin and eosin staining of wild type and SLIRP knockout testis shows there to be no overt difference in morphology between the genotypes.
(TIFF)

Figure S4 **Testosterone, FSH and LH levels do not differ between SLIRP genotypes.** A) Testosterone, B) follicular stimulating hormone (FSH) and C) luteinizing hormone (LH) levels are not significantly different between wild type (WT) and homozygous SLIRP knockout (KO) mice when compared by ANOVA. Assessment of testosterone levels was performed by liquid chromatography tandem mass spectrometry (LC/MS) at the Biochemistry Department at Royal Perth Hospital. The liquid chromatography was performed using a Waters Acquity Model UPH and a VisionHT C18 column (1.5 µm, 50×2.0 mm) fitted with a C18 guard (Grace Davison Discovery Sciences). The mass spectrometer was a Waters Micromass Quattro Premier XE. The application manager software use was QUANLynx v 4.1. FSH and LH concentrations were determined using heterologous radioimmunassays as described previously in Kennedy *et al.*, (2006) A repository of ENU mutant mouse lines and their potential for male fertility research. *Mol Hum Reprod*. Dec;11(12):871–880.
(TIFF)

Figure S5 **Sperm isolated from wild type SLIRP mice have normal progressive motility.** This image is the first frame of Movie S1, recorded following 30 min of capacitation at 37°C in human tubal fluid.
(TIFF)

Figure S6 **Sperm isolated from SLIRP KO mice have reduced progressive motility.** This may be observed when videos of sperm isolated from the epididymis of wild type (see Movie S1) and SLIRP knockout (this figure) mice are compared. This image is the first frame of Movie S2, recorded following 30 min of capacitation at 37°C in human tubal fluid.
(TIFF)

Figure S7 **Annulus and mitochondrial disruptions in SLIRP KO sperm.** Additional electron micrographs of wild type (A & B) and SLIRP knockout (KO) sperm (C & D). The annulus of both the wt and KO sperm are highlighted (white arrows). The cisternae of KO mouse mitochondria are less defined and their matrices are more electron dense that those of wt mice. Electron microscopy was performed as described in Arsov T, *et al.* (2006) Fat aussie-a new Alstrom syndrome mouse showing a critical role for ALMS1 in obesity, diabetes, and spermatogenesis. *Mol Endocrinol* 20(7):1610–1622.
(TIFF)

Movie S1 **Sperm isolated from wild type SLIRP mice have normal progressive motility.** Video of sperm isolated from the epididymis of wild type SLIRP mice recorded following 30 min of capacitation at 37°C in human tubal fluid.
(MOV)

Movie S2 **Sperm isolated from SLIRP KO mice have reduced progressive motility.** Video of sperm isolated from the epididymis of SLIRP knockout mice recorded following 30 min of capacitation at 37°C in human tubal fluid.
(MOV)
