# Supplementary figures and images for: Loss of the Nuclear Receptor Corepressor SLIRP Compromises Male Fertility
Source: PLoS One. 2013 Aug 15;8(8):e70700. doi: 10.1371/journal.pone.0070700 (PMC3744554; doi:10.1371/journal.pone.0070700)

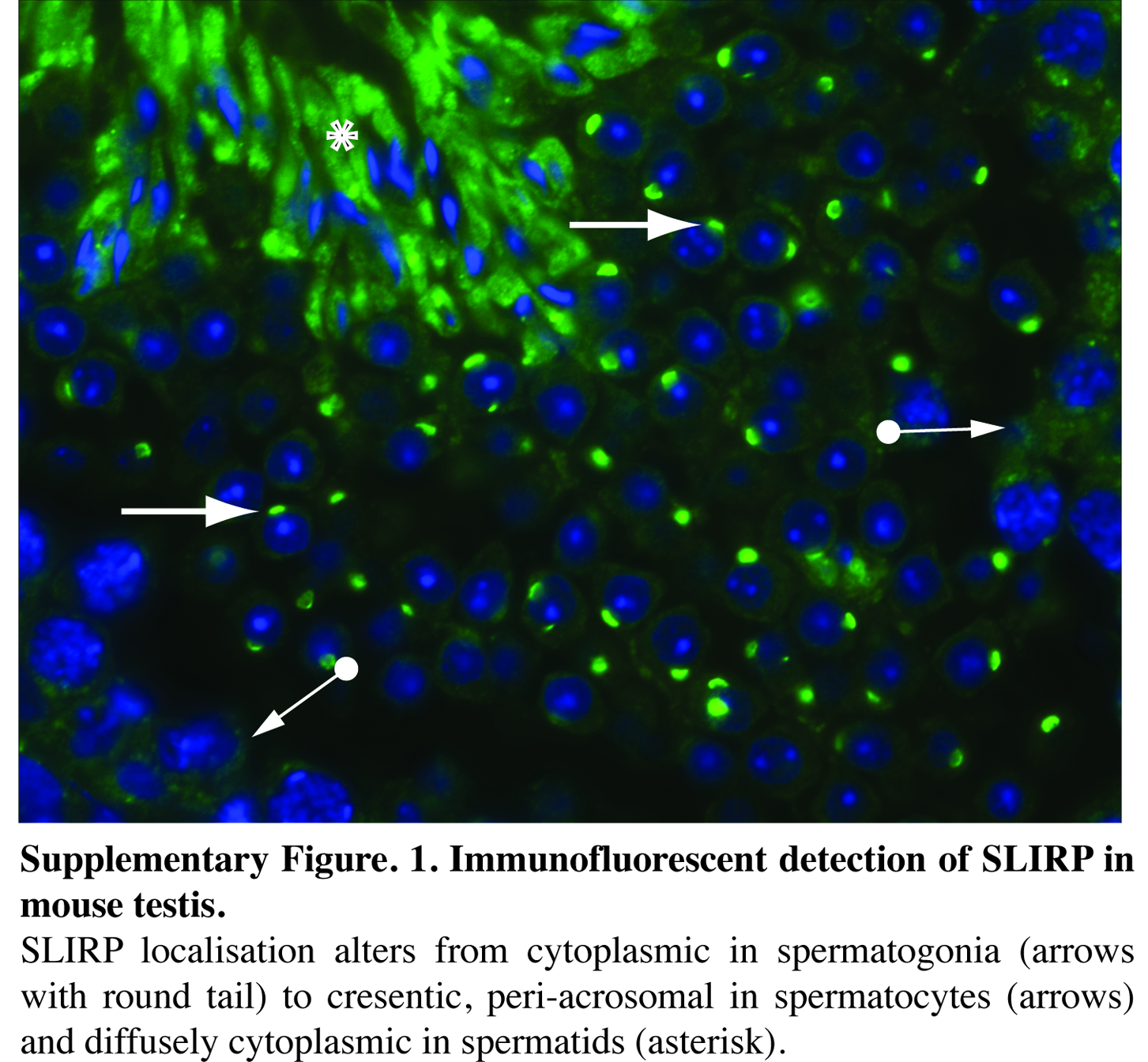

Supplement: Figure S1 — Immunofluorescent detection of SLIRP in mouse testis. SLIRP localisation alters from cytoplasmic in spermatogonia (arrows with round tail) to cresentic, peri-acrosomal in spermatocytes (arrows) and diffusely cytoplasmic in spermatids (asterisk). (TIFF) [file pone.0070700.s001.tif]

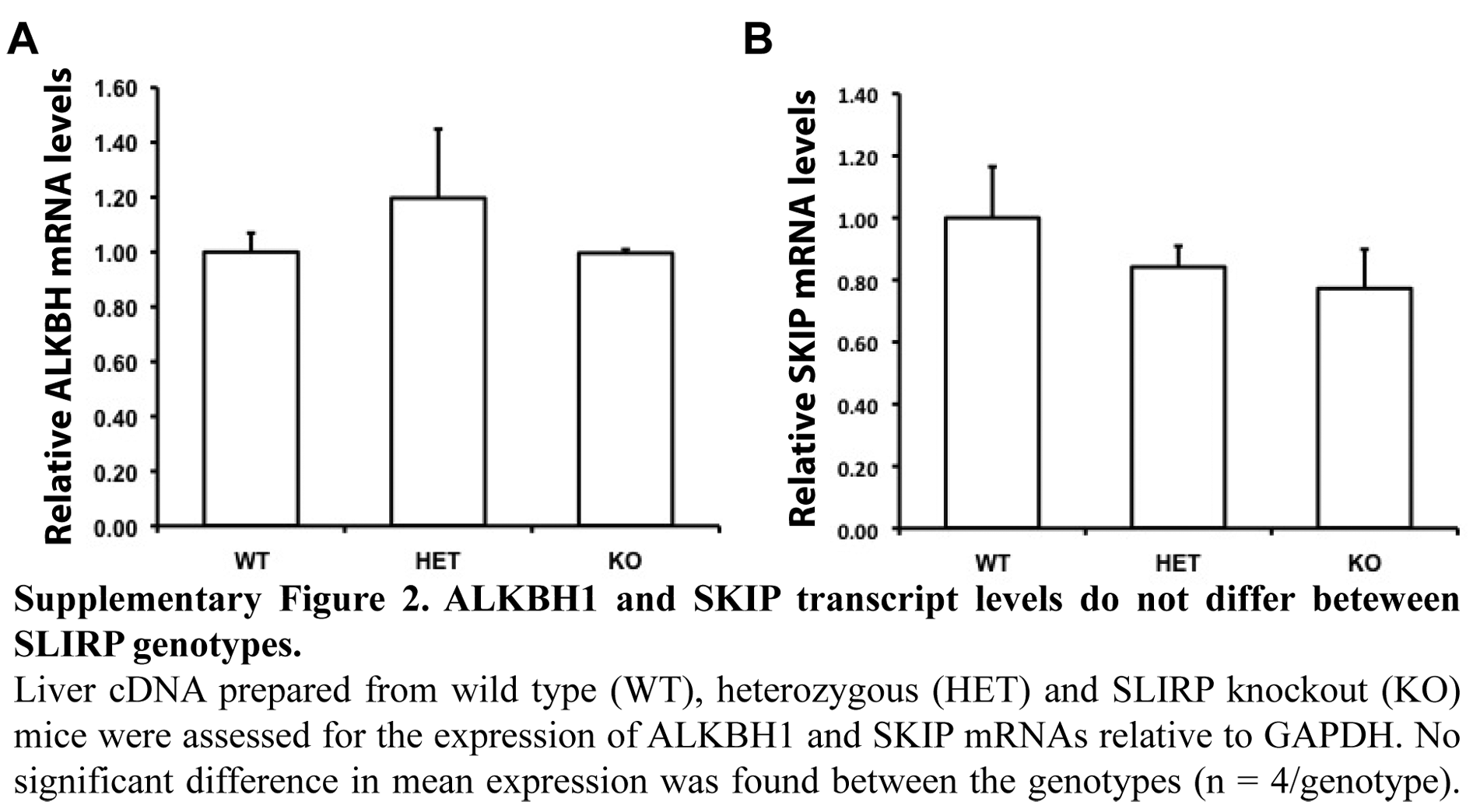

Supplement: Figure S2 — ALKBH1 and SKIP transcript levels do not differ beteween SLIRP genotypes. Liver cDNA prepared from wild type (WT), heterozygous (HET) and SLIRP knockout (KO) mice were assessed for the expression of ALKBH1 and SKIP mRNAs relative to GAPDH. No significant difference in mean expression was found between the genotypes (n = 4/genotype). (TIFF) [file pone.0070700.s002.tif]

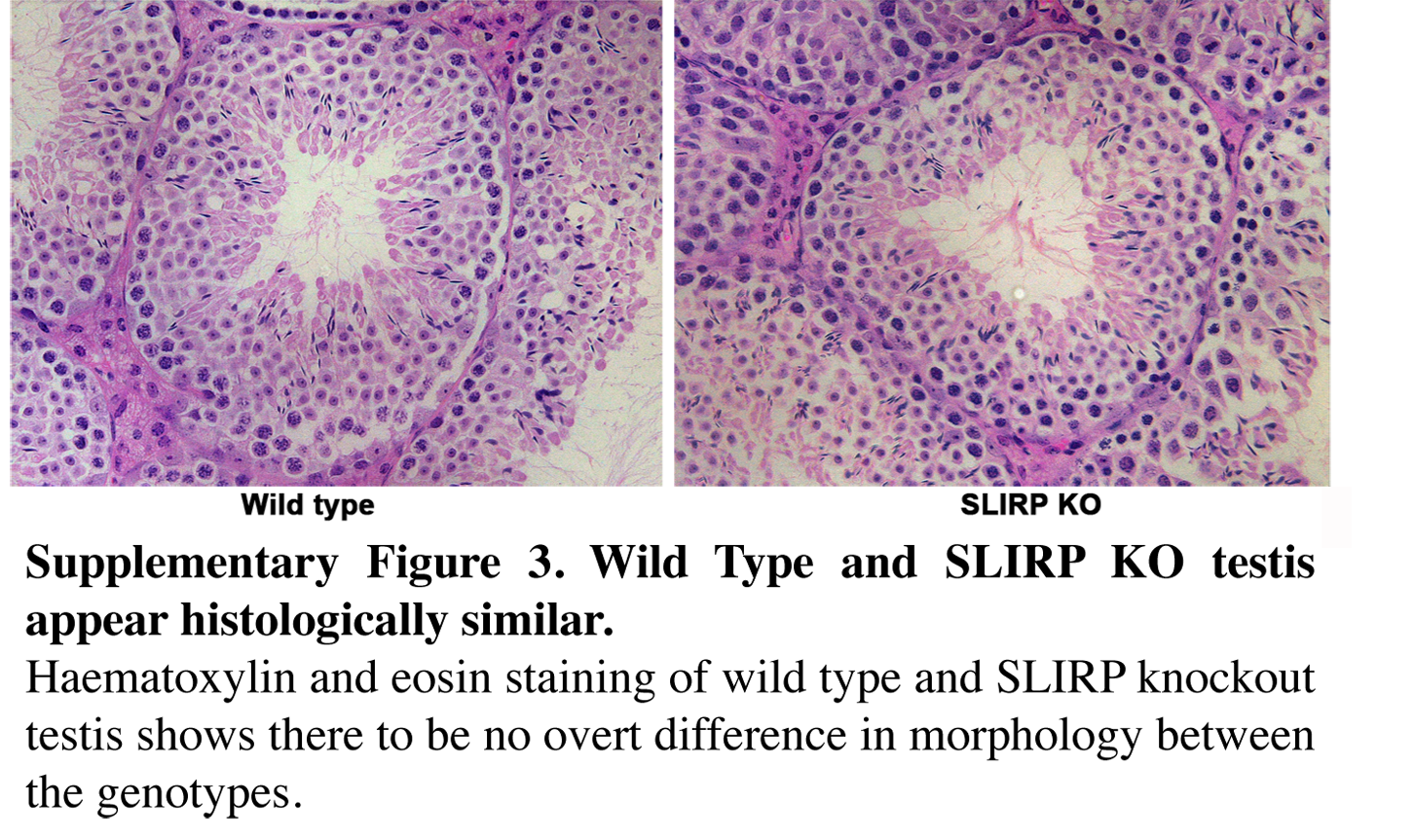

Supplement: Figure S3 — Wild Type and SLIRP KO testis appear histologically similar. Haematoxylin and eosin staining of wild type and SLIRP knockout testis shows there to be no overt difference in morphology between the genotypes. (TIFF) [file pone.0070700.s003.tif]

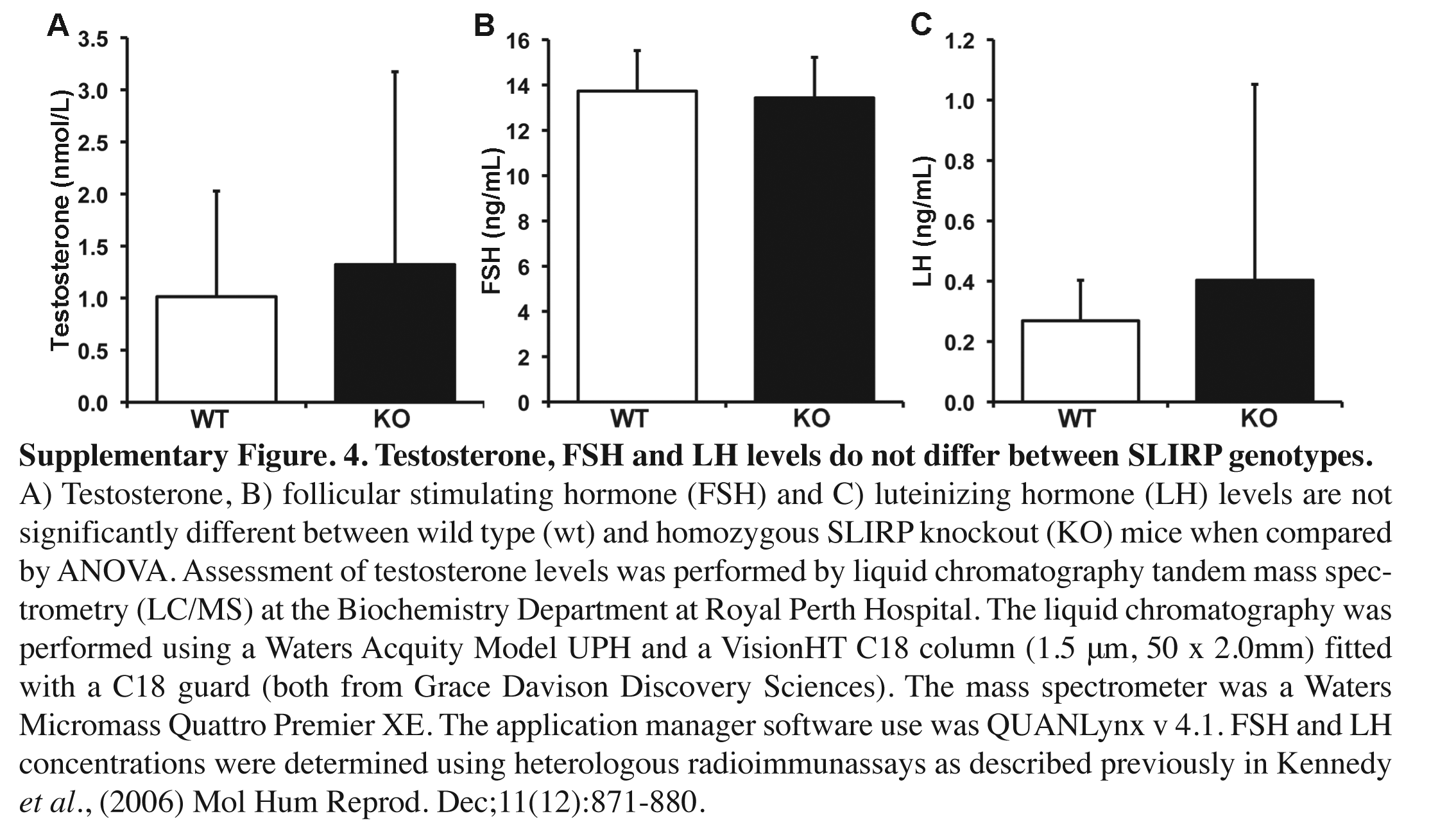

Supplement: Figure S4 — Testosterone, FSH and LH levels do not differ between SLIRP genotypes. A) Testosterone, B) follicular stimulating hormone (FSH) and C) luteinizing hormone (LH) levels are not significantly different between wild type (WT) and homozygous SLIRP knockout (KO) mice when compared by ANOVA. Assessment of testosterone levels was performed by liquid chromatography tandem mass spectrometry (LC/MS) at the Biochemistry Department at Royal Perth Hospital. The liquid chromatography was performed using a Waters Acquity Model UPH and a VisionHT C18 column (1.5 µm, 50×2.0 mm) fitted with a C18 guard (Grace Davison Discovery Sciences). The mass spectrometer was a Waters Micromass Quattro Premier XE. The application manager software use was QUANLynx v 4.1. FSH and LH concentrations were determined using heterologous radioimmunassays as described previously in Kennedy et al., (2006) A repository of ENU mutant mouse lines and their potential for male fertility research. Mol Hum Reprod. Dec;11(12):871–880. (TIFF) [file pone.0070700.s004.tif]

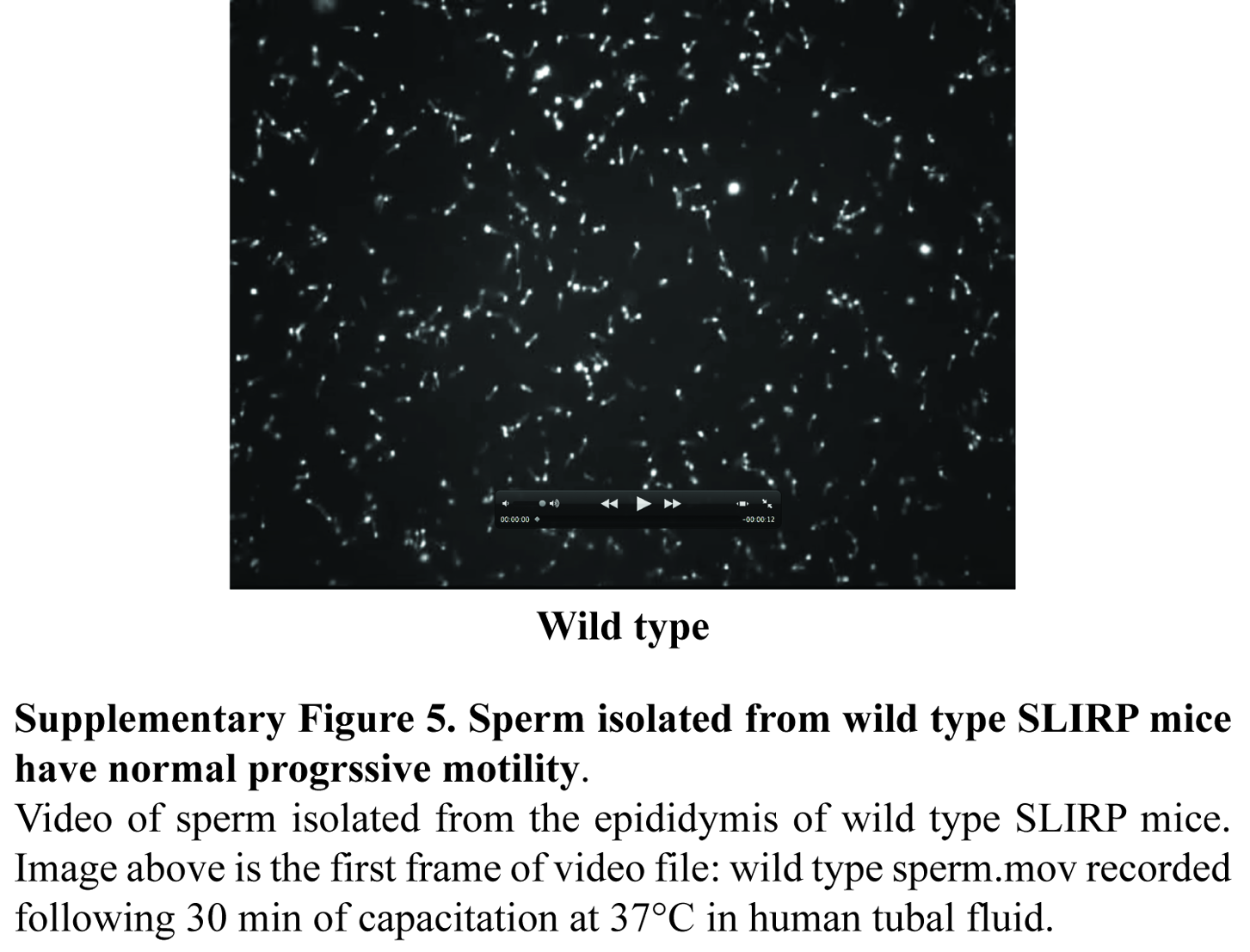

Supplement: Figure S5 — Sperm isolated from wild type SLIRP mice have normal progressive motility. This image is the first frame of Movie S1, recorded following 30 min of capacitation at 37°C in human tubal fluid. (TIFF) [file pone.0070700.s005.tif]

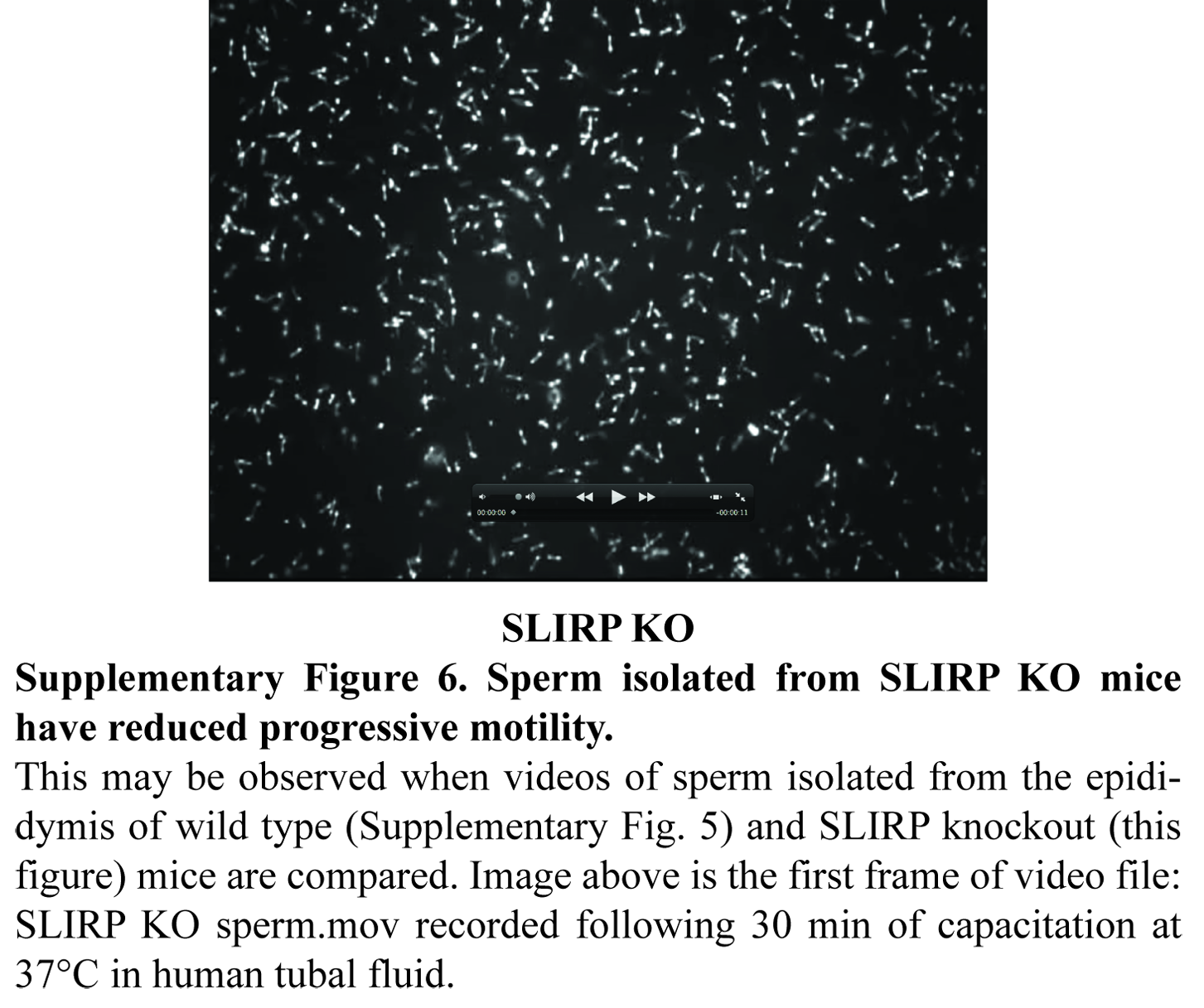

Supplement: Figure S6 — Sperm isolated from SLIRP KO mice have reduced progressive motility. This may be observed when videos of sperm isolated from the epididymis of wild type (see Movie S1) and SLIRP knockout (this figure) mice are compared. This image is the first frame of Movie S2, recorded following 30 min of capacitation at 37°C in human tubal fluid. (TIFF) [file pone.0070700.s006.tif]

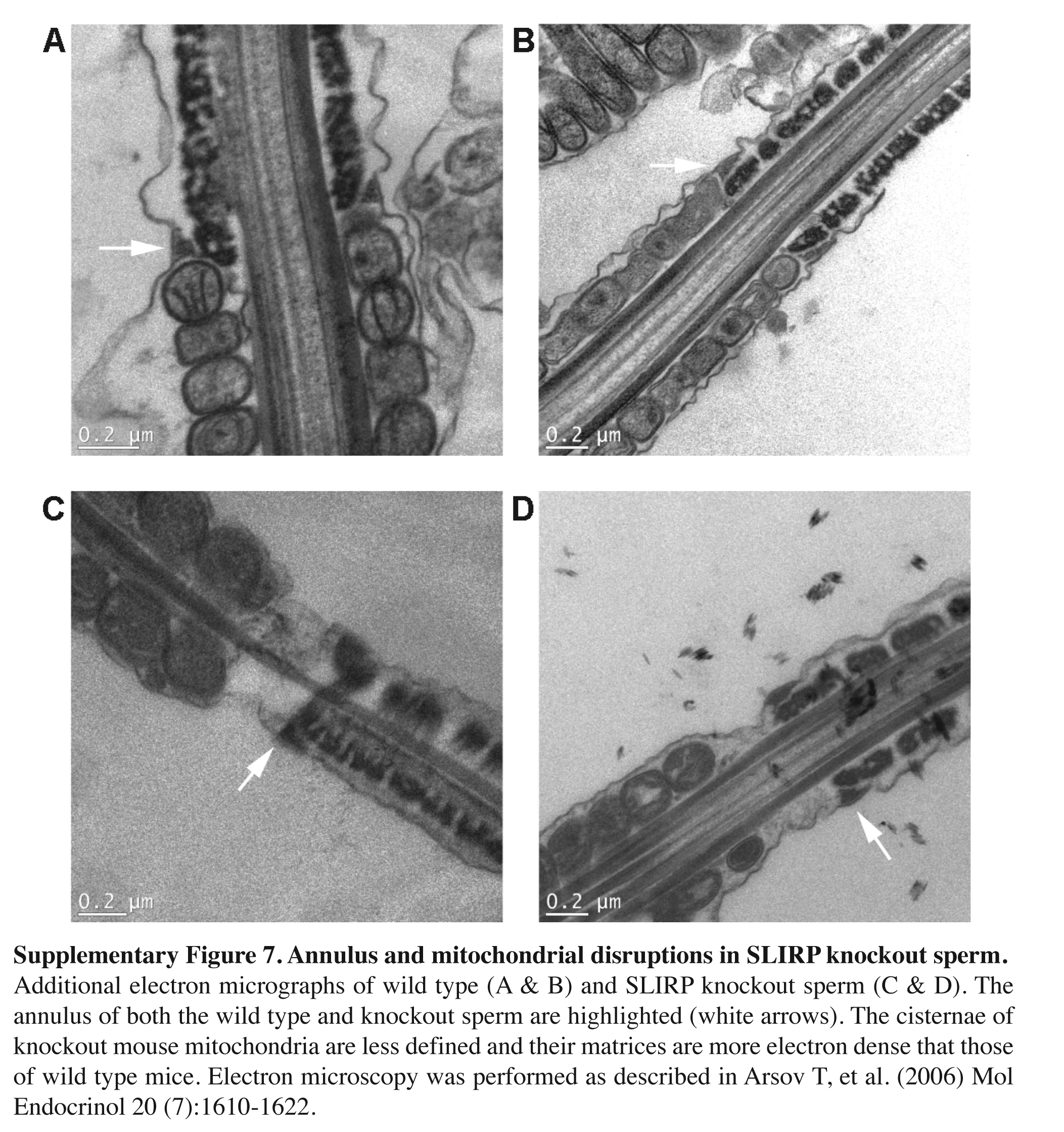

Supplement: Figure S7 — Annulus and mitochondrial disruptions in SLIRP KO sperm. Additional electron micrographs of wild type (A & B) and SLIRP knockout (KO) sperm (C & D). The annulus of both the wt and KO sperm are highlighted (white arrows). The cisternae of KO mouse mitochondria are less defined and their matrices are more electron dense that those of wt mice. Electron microscopy was performed as described in Arsov T, et al. (2006) Fat aussie-a new Alstrom syndrome mouse showing a critical role for ALMS1 in obesity, diabetes, and spermatogenesis. Mol Endocrinol 20(7):1610–1622. (TIFF) [file pone.0070700.s007.tif]
